# Development of Genome-Wide Insertion and Deletion Polymorphism Markers from Next-Generation Sequencing Data in Rice

**DOI:** 10.1186/s12284-015-0063-4

**Published:** 2015-08-14

**Authors:** Jian Liu, Jingwei Li, Jingtao Qu, Shuangyong Yan

**Affiliations:** Maize Research Institute, Sichuan Agricultural University, Chengdu, Sichuan 611130 China; Key Laboratory of Biology and Genetic Improvement of Maize in Southwest Region, Ministry of Agriculture, Chengdu, Sichuan 611130 China; Tianjin Crops Institute, Tianjin, 300112 China

**Keywords:** Rice, Insertion and deletion, Polymorphism markers, Next-generation sequence

## Abstract

**Background:**

Next-generation sequencing technologies enable the re-sequencing of a large number of genomes and provide an unprecedented opportunity to discover numerous DNA polymorphisms throughout the genome of a species. As the second most abundant form of genetic variation, InDels, with characteristics of co-dominance, multiple alleles and high stability and density and that are easy to genotype, have received an increasing amount attention.

**Results:**

In this work, a total of 2,329,544 InDels were identified in 1767 rice genomes; these InDels were dispersed across all 12 rice chromosomes, with one InDel marker found, on average, every 160.22 bp. There were 162,380 highly polymorphic InDels with a polymorphism information content (PIC) ≥ 0.5, contributing 1.81 % to the unique primer set. Of these highly polymorphic InDels, we also selected InDels with major allele differences (the size difference between the most and second most frequent alleles) ≥ 3 bp or 8 bp for primer design, which provided a more flexible choice for researchers. Finally, we experimentally validated 100 highly polymorphic InDels for accuracy and polymorphism. The PCR results showed that the accuracy of the InDel markers was 95.70 %, while the average PIC value was 0.56, with a range of 0.19 to 0.78; the average allele number was 3.02, with a range of 2 to 5.

**Conclusions:**

Our genome-wide and easily used InDel markers with high polymorphism and density in both cultivated and wild rice will undoubtedly have practical implications in rice marker-assisted breeding and will also meet the need of fine-scale genetic mapping in map-based rice gene cloning.

**Electronic supplementary material:**

The online version of this article (doi:10.1186/s12284-015-0063-4) contains supplementary material, which is available to authorized users.

## Background

Rice (*Oryza sativa* L.), which is grown worldwide and is an important food crop for more than half of the world’s population, is considered to be the monocot model plant for molecular genetic studies. To be grown successfully under a variety of climatic conditions, breeders maintain rice with high genetic diversity. Molecular markers are useful tools for genetic research and breeding, including genotype fingerprinting, genetic diversity analysis, phylogenetic analysis, map-based gene cloning, variety identification and marker-assisted breeding (McCouch et al. 1997; Joshi et al. 2001; Nagaraju et al. 2002; Ni et al. 2002). A large number of molecular markers have been used in rice, such as restriction fragment length polymorphisms (RFLPs), simple sequence repeat (SSR) markers, single nucleotide polymorphisms (SNPs) and insertions-deletions (InDels) (Akagi et al. 1996; Panaud et al. 1996; Temnykh et al. 2001; McCouch et al. 2002; Nasu et al. 2002; Project IRGS 2005; Ren et al. 2005; Chen et al. 2011). SSRs and SNPs are the two most widely used molecular markers in rice. SSR markers are co-dominant, multi-allelic, easy to use and inexpensive; thus, they have been the most widely used markers in rice for a long period of time (Tautz 1989; McCouch et al. 2002). SNP markers occur at a much higher density in the genome and are amenable to high-throughput methods, such as genotyping arrays (Rafalski 2002). It is worth noting that the low density of SSRs is a major obstacle for their application; large-scale use of SNPs is restricted by less information and alleles as well as high technical or equipment demands. However, on average, the cost of high-density SNP genotyping is relatively low.

As the second most common type of polymorphism and the most abundant structural variant (Mullaney et al. 2010), InDel markers provide a higher density than traditional SSR markers and utilize the same experimental procedure as that used for SSR markers. Because InDel markers have more practical value for laboratories without the infrastructure to perform SNP genotyping (Liu et al. 2013) and because they occur more frequently than SSR markers in the rice genome and use the same experimental approaches that are routinely used for SSR markers, they have received an increasing amount of attention. Recently, InDel markers have been successfully used for genetic studies in wheat (Raman et al. 2006), rice (Hayashi et al. 2006; Ji et al. 2010; Liu et al. 2012), citrus (García-Lor et al. 2012), Arabidopsis (Hou et al. 2010; Păcurar et al. 2012) and natural populations of dogs (Väli et al. 2008). Specifically, for rice, 50 InDel markers developed by Shen et al. (2004) have successfully been used to distinguish the Basmati rice variety from other fragrant rice varieties (Steele et al. 2008). In addition, InDel markers have also been used for the classification of rice indica and japonica subspecies and to examine their relationships regarding evolution (Lu et al. 2009; Liu et al. 2012).

Based on first-generation (Sanger) sequencing, the genomic sequences of two inbred rice cultivars, japonica cv. Nipponbare and indica cv. 9311, were released during the past decade (Goff et al. 2002; Yu et al. 2002). The emergence of next-generation sequencing (NGS) technologies has enabled more efficient re-sequencing of a large number of genomes at a significantly lower cost than ever before and has provided the opportunity to survey high-throughput genotyping and large-scale genetic variation (Weigel and Mott 2009). A massive number of InDel polymorphisms between highly homologous genomes in rice have been identified more efficiently and economically than ever before by the NGS technique (Arai-Kichise et al. 2011). An alignment of shotgun-sequenced contigs of 9311 and the draft genome assembly of Nipponbare revealed 24,557 single-base InDel polymorphisms between the two subspecies, resulting in a polymorphism rate of 0.11 InDels/kb across the whole genome (Feltus et al. 2004). Shen et al. (2004) has constructed a genome-wide DNA polymorphism database using the genome of Nipponbare, a cultivar of japonica, and 9311, a cultivar of indica, which contains 479,406 InDels (Shen et al. 2004).

A large amount of InDel markers have been developed in rice in previous studies, but they lack polymorphism information or allele frequency in different populations or subpopulations. In this article, we collected the re-sequencing data of 1767 worldwide rice varieties from publicly available genomic sequence information to develop genome-wide InDel polymorphism markers through a computational strategy called electronic-PCR (e-PCR) (Schuler 1997); in addition, we experimentally validated some InDel polymorphisms. Thus, the genome-wide and easily used InDel markers with polymorphism information content (PIC) ≥ 0.5 and major allele differences (size difference between the most and second most frequent alleles) ≥ 3 bp or 8 bp, which were clustered by the major groups of *O. sativa (Japonica and Indica)*, *O. rufipogon* and *O. nivara*, will have significant practical implications in rice breeding.

## Results

### Identification and Distribution of the Unique Primer

Using the DNA sequences of the Nipponbare reference genome as the template, we designed 18,662,247 pairs of e-PCR primers based on the sliding window technique, which included 7,738,481 pairs in the intergenic region and 10,923,766 pairs in the genic region, accounting for 41.47 % and 58.53 % of the genic region, respectively. Of the total e-PCR primers, 8,995,927 primer pairs were mapped to the unique genomic regions in both Nipponbare and 9311 simultaneously with a proportion of 48.20 %, which included 3,560,733 pairs in the intergenic region and 5,435,194 pairs in the genic region, accounting for 39.58 % and 60.42 %, respectively. Based on data from Table 1, the density of these unique primers in different genomic regions varied and were ranked in descending order as follows: intron, 3′-UTR (3′-untranslated region), 5′-UTR (5′-untranslated region), TSS_up_0.5 kb (0.5 kb upstream of the transcription start site), intergenic, TES_down_0.5 kb (0.5 kb downstream of the transcription end site) and CDS (coding determining sequences).

**Table 1:** Distribution of the primers and InDels in various genomic regions

Genome Region	Total Primer^a^	Unique Primer^b^			Unique Primer Set^c^	InDel^d^			Highly Polymorphic INDEL^e^		
	Count	Count	Density^f^	Proportion (%)^g^	Count	Count	Density^f^	Proportion (%)^h^	Count	Density^f^	Proportion (%)^i^
TSS_up_0.5 kb	1,372,500	659,382	23.63	48.04	655,277	227,877	8.17	34.78	17,555	0.63	2.68
5′-UTR	424,611	225,331	26.66	53.07	223,737	72,931	8.63	32.60	6061	0.72	2.71
3′-UTR	562,748	435,385	27.64	77.37	435,014	104,248	6.62	23.96	6295	0.40	1.45
CDS	3,844,531	1,545,879	17.48	40.21	1,536,689	158,448	1.79	10.31	4640	0.05	0.30
Intron	3,571,402	2,106,294	39.90	58.98	2,101,227	483,308	9.16	23.00	33,985	0.64	1.62
TES_down_0.5 kb	1,329,621	643,369	23.06	48.39	641,322	192,099	6.89	29.95	13,883	0.50	2.16
Intergenic	7,738,481	3,560,733	23.42	46.01	3,542,446	1,130,446	7.44	31.91	82,495	0.54	2.33
Total	18,662,247	8,995,927	24.10	48.20	8,955,862	2,329,724	6.24	26.01	162,380	0.44	1.81

^a^Total primers in various genomic regions

^b^Primer pairs simultaneously mapped to unique genomic regions in both Nipponbare and 9311

^c^A set of unique primers with the e-PCR products in twenty or more than twenty rice genomes

^d^InDels with PIC > 0

^e^InDels with PIC ≥ 0.5

^f^Density was defined as the corresponding primer or InDel number every 1000 bp

^g^Proportion of unique primers in the total primers

^h^Proportion of InDels in the unique primer set

^i^Proportion of highly polymorphic InDels in the unique primer set

In addition, we also compared the distribution of the unique primers on the 12 rice chromosomes, and the results showed that the count of unique primers on each chromosome was generally consistent with the corresponding chromosome length. The smallest distribution was observed on chromosome 10 (496,144), while the largest distribution was on chromosome 1 (1,175,638). The average density of unique primers dispersed along the 12 rice chromosomes varied from the lowest (19.39 primer pairs/kb, or one primer pair every 51.57 bp) on chromosome 11 to the highest (28.75 primer pairs/kb, or one primer pair every 34.79 bp) on chromosome 3, with an average size of 24.10 primer pairs/kb (one primer pair every 41.49 bp) across all 12 chromosomes (Table 2).

**Table 2:** Distribution of the unique primers and InDels on rice chromosomes

Chromosome	Chromosome length (bp)	Unique primer^b^		InDel^d^	
		Count	Density^f^	Count	Density^f^
1	43270923	1,175,638	27.17	299,457	6.92
2	35937250	1,011,186	28.14	259,989	7.23
3	36413819	1,046,792	28.75	262,345	7.20
4	35502694	777,218	21.89	196,803	5.54
5	29958434	728,667	24.32	190,707	6.37
6	31248787	754,761	24.15	200,616	6.42
7	29697621	733,083	24.68	191,446	6.45
8	28443022	604,746	21.26	158,366	5.57
9	23012720	555,769	24.15	146,065	6.35
10	23207287	496,144	21.38	129,305	5.57
11	29021106	562,780	19.39	150,050	5.17
12	27531856	549,143	19.95	144,395	5.24
Total	373245519	8,995,927	285.24	2,329,544	74.04
Average	31103793.25	749,660.58	24.10	194,128.67	6.24

^b^Primer pairs simultaneously mapped to unique genomic regions in both Nipponbare and 9311

^d^InDels with PIC > 0

^f^Density was defined as the corresponding primer or InDel number every 1000 bp

### InDel Identification in Rice Varieties

Based on the next-generation sequencing data from 1765 rice varieties, a total of 7,100,998,643 publicly sequenced reads were downloaded from the NCBI (National Center for Biotechnology Information) website, with an average length of 86.56 bp; the sequencing depth ranged from 0.03× (GP133) to 118.42× (Oryza-brachyantha), with an average depth of 1.89× (Additional file 1: Table S1). Using e-PCR to align 8,995,927 unique primer pairs against publicly sequenced reads of rice varieties, the results showed that the average number of e-PCR products was 1,950,638, with a proportion of 21.68 % (Additional file 2: Table S2), which ranged from 10,631 in Bengal (0.12 %) to 8,764,237 in Omachi (97.42 %). A set of 8,955,862 unique primers had e-PCR products in twenty or more than twenty rice genomes, contributing more than 99.55 % to the total unique primers; then, we identified 2,329,544 InDels based on this set of unique primers, contributing 26.01 % to the unique primer set (Table 1).

### InDel Distribution in Rice Genomes

The 2,329,544 InDels were almost evenly distributed across all 12 chromosomes, but the distribution of InDels varied from the smallest amount (129,305) on chromosome 10 to the largest amount (299,457) on chromosome 1. The average density was 6.24 InDels/kb, with a range of 5.17 to 7.23 InDels/kb on 12 chromosomes. The lowest density was on chromosome 11, and the highest density was on chromosome 2 (Table 2). Subsequently, we also looked into the details of InDels in various genomic regions and found that InDels were most abundant in the intergenic region, followed, in order, by intron, TSS_up_0.5 kb, TES_down_0.5 kb, CDS, 3′-UTR and 5′-UTR (Table 1). Thus, the proportion of InDels distributed in the intergenic region was 48.52 %, and the remaining 51.48 % were distributed in the genic region.

**Fig. 1:**
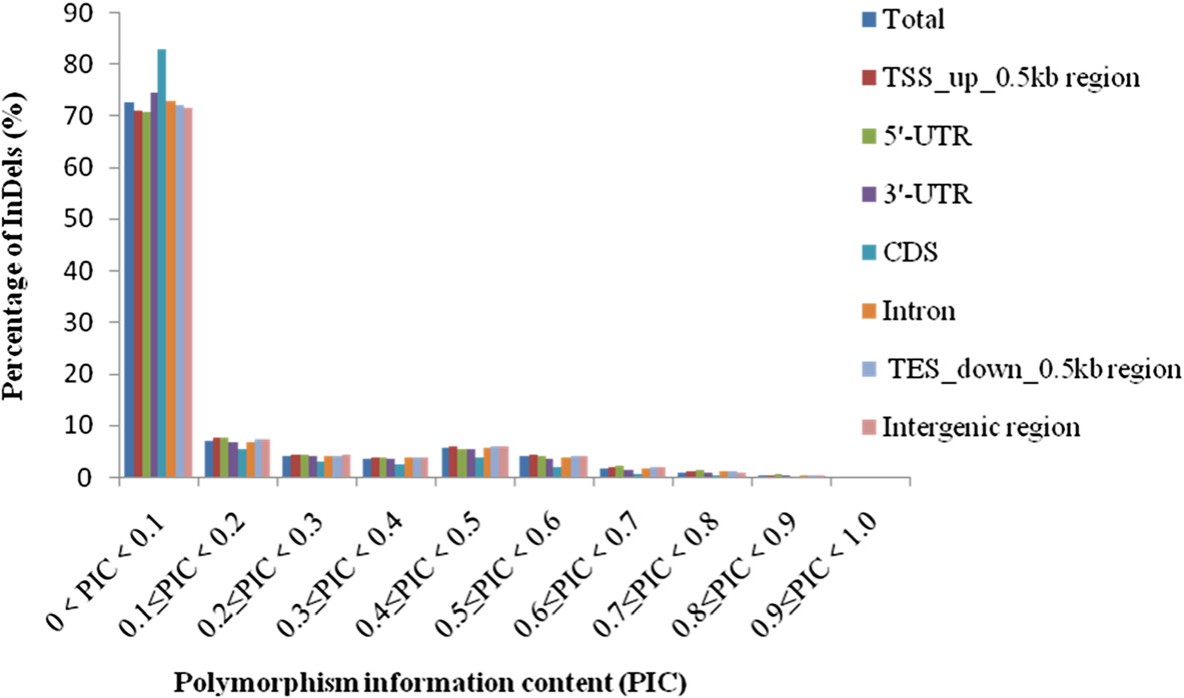
Distribution of InDels in various genomic regions

The number of InDels decreased with increasing PIC value, and most (1,690,818) had a PIC value with a range from 0 to 0.1, contributing more than 70 % to the total InDels (Fig. 1). With respect to the polymorphism rate (the proportion of InDels in the unique primer set), we concluded that InDels in TSS_up_0.5 kb were the most abundant, followed by 5′-UTR, intergenic, TES_down_0.5 kb, 3′-UTR, intron and CDS (Table 1). In particular, CDS had the lowest polymorphic rate (10.31 %), with the highest (82.82 %) PIC values, varying from 0 to 0.1, while TSS_up_0.5 kb had the highest polymorphic rate (34.78 %) and the fewest (70.84 %) PIC values varying from 0 to 0.1.

**Fig. 2:**
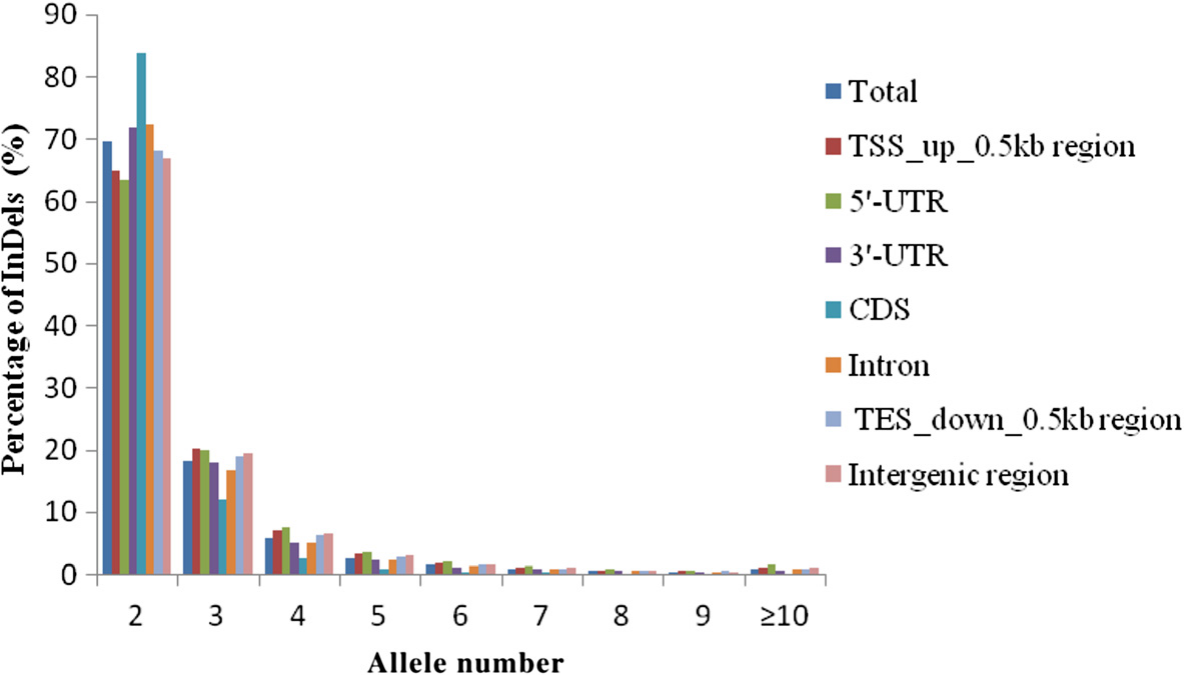
Distribution of allele number in various genomic regions

**Fig. 3:**
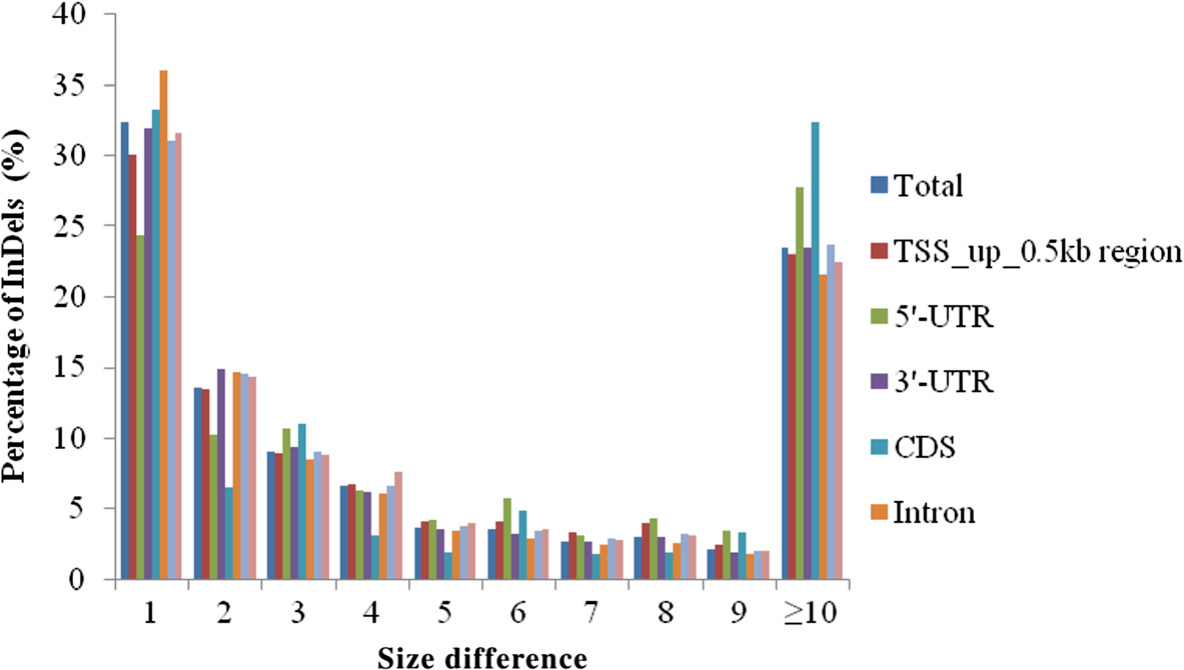
Distribution of InDel size difference in various genomic regions

The number of alleles generated for all InDels varied from 2 to 116, with an average of 2.82. For the InDels distributed in various genomic regions, most (69.66 %) had two alleles, 18.10 % had three alleles, and only a few markers had nine alleles (Fig. 2). Interestingly, for two alleles, CDS had the highest percentage of InDels (83.70 %) and 5′-UTR had the lowest percentage of InDels (63.32 %), but for the remaining allele number, the highest amount of InDels were almost all distributed in the 5′-UTR and the lowest amount were all in the CDS. We also analyzed the polymorphism of the InDel size in various genomic regions and found that the InDel size difference between the shortest and the longest varied from 1 to 214 bp. The majority of the InDel markers (76.50 %) had less than 10-bp size difference: 32.42 % had a size difference of 1 bp, 13.53 % had a size difference of 2 bp, 9.03 % had a size difference of 3 bp, 6.57 % had a size difference of 4 bp, 3.64 % had a size difference of 5 bp, 3.56 % had a size difference of 6 bp, 2.67 % had a size difference of 7 bp, 2.96 % had a size difference of 8 bp and 2.11 % had a size difference of 9 bp. Only 23.50 % of the markers had a size difference of more than 10 bp. Thus, the size differences of 1 and 2 bp made up a higher percentage (45.95 %) than the size differences from 3 bp to 9 bp (30.54 %). For various genomic regions, except the CDS region, all had a declining percentage with increasing InDel size difference (Fig. 3).

### Primer Design for New InDel Markers

For different resolutions of polyacrylamide gel electrophoresis and agarose gel electrophoresis, two sets of highly polymorphic InDel markers were selected from 162,380 highly polymorphic InDels with PIC ≥ 0.5. One set of 41855 InDels with major allele difference ≥ 3 bp and PCR product lengths of 60–100 bp was selected for polyacrylamide gel electrophoresis (Additional file 3: Table S3), while the other set of 14428 InDels with a major allele difference ≥ 8 bp and PCR product lengths of 150–300 bp was selected for agarose gel electrophoresis (Additional file 4: Table S4). The exact positions of these InDels, as well as information on the PCR-based primers and PIC are presented in Table S3 and Table S4. These positions would be very useful for primer selection in actual practice, and researchers could also design primers from the tag sequences surrounding the InDels. In addition, major allele frequencies within the major groups of *O. sativa (Japonica and Indica)*, *O. rufipogon* and *O. nivara* and the gene ID and annotation, if available, are included in Table S3 and Table S4 as well. Finally, we also provided an ontological analysis of the genes affected by I with PIC ≥ 0.5 and a major allele difference ≥3 bp in the CDS region (Additional file 5: Table S5); these characteristics made these InDels valuable for molecular breeding applications.

### Experimental Validation

A set of 100 InDel markers that were evenly spread across the rice genome were selected, and primers were designed for experimental validation (Additional file 6: Table S6). The 100 InDel markers were developed by e-PCR based on the InDel polymorphisms between the Nipponbare and 9311 reference sequences; thus, these markers should theoretically be polymorphic between Nipponbare and 9311. To test their accuracy, we amplified the genomic DNA of Nipponbare and 9311 by PCR. The PCR results showed that only seven primer pairs could not simultaneously generate PCR products from the genomic DNA of both Nipponbare and 9311, and 89 primer pairs were polymorphic between Nipponbare and 9311; thus, the PCR success rate was 93 %, while the accuracy of InDel markers was 95.70 %.

**Fig. 4:**
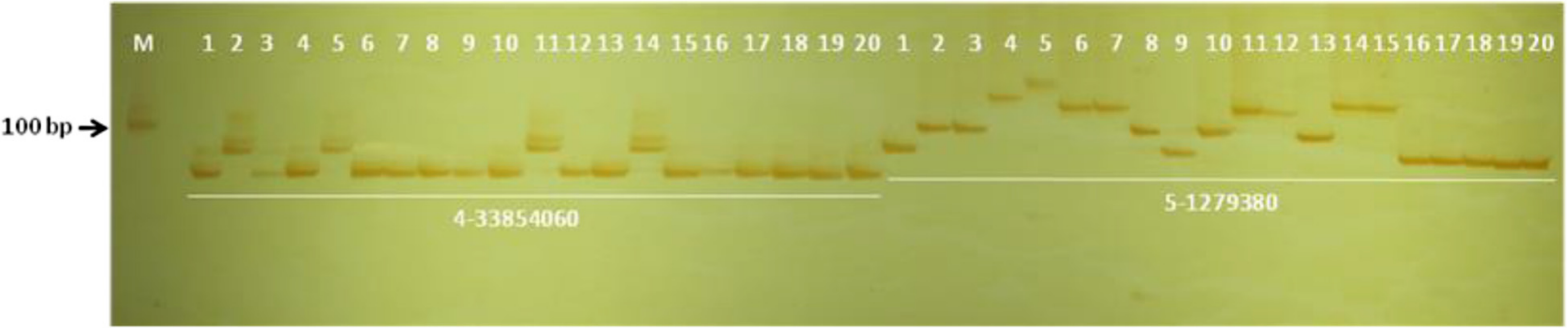
Experimental validation for two InDels on 4-33854060 and 5-1279380. PCR product from lane1 to lane20 was Nipponbare, 9311, zaomadao, SRSye, beixiang7, 60kang, 72gan, 955R, T116, sanpang76, R1318, nanhui511, guiyangai49, qingnongai, huangxinzhan, yanjing2, yandao8, xudao3, huaidao13 and wuyunjing3, respectively. (The number below horizontal line showed chromosome and physical position. M: Marker DL2000)

To test their polymorphisms in other rice varieties, we further analyzed them by genomic DNA PCR amplification in a panel of 20 rice cultivars, including Nipponbare and 9311, using the same set of InDel markers plus 100 pairs of SSR primers for comparison (Additional file 7: Table S7; Fig. 4). For the InDel markers, their average accuracy was approximately 98.2 % in 20 rice cultivars, while the average PIC value was 0.56, with a range of 0.19 to 0.78, and the average allele number was 3.02, with a range of 2 to 5, using the genomic DNA of 20 rice cultivars as the template for PCR amplification. However, the PIC value varied from 0.50 to 0.95, with an average of 0.78, and the allele number varied from 2 to 34, with an average of 12.25, using the re-sequencing data of 1765 rice varieties as the template for e-PCR. For 100 pairs of SSR primers, the PIC value ranged from 0 to 0.78, with an average of 0.35, and the allele number ranged from 1 to 5, with an average of 2.17 (Additional file 8: Table S8).

## Discussion

The emergence of next-generation sequencing technologies has enabled more efficient genome sequencing at a significantly lower cost than ever before and has provided the community with the opportunity to find genome-wide DNA polymorphisms for genetic studies. In this article, we examined 1767 rice genomes, representing a much wider sample than has previously been used, and detected a high number of InDels across the rice genome, which was nearly three- and four-fold more than in previous work (Xu et al. 2012; Huang et al. 2012); thus, we developed the most broadly representative high-density InDel markers, with one InDel every 160 bp along the rice genome, which was nearly 65-fold of that of genes (Project IRGS 2005), one-thousand times denser than SSR and six times denser than other InDels developed in previous studies (Shen et al. 2004). Accordingly, these genome-wide InDel polymorphism markers could meet the need of fine-scale genetic mapping in map-based cloning of rice genes. Notably, more than half of the InDels are distributed in genic regions, and these InDels are considered to be genic molecular markers or functional markers (Varshney et al. 2007) that are superior to random DNA markers, such as RFLPs, SSRs and AFLPs, because of complete linkage with trait locus alleles (Andersen and Lübberstedt 2003). In addition, genic molecular markers have provided opportunities to examine functional diversity, and with the development of more genic molecular markers in major crop species, genetic diversity studies will become more meaningful.

Except for high density, high polymorphism is the most important evaluation factor for marker development. Our genome-wide InDel polymorphism markers also contains highly polymorphic InDel markers (PIC ≥ 0.5), and PCR validation has proved our markers to be highly polymorphic. The average polymorphism and allele number of the InDels are both higher than those of SSR in PCR validation, but lower than those in e-PCR. This is mainly because the gel resolution is lower than e-PCR and because e-PCR can detect even one base difference. In addition, the detection efficiency of gel electrophoresis not only requires a size difference but also demands an amplicon length. The detection efficiency is enhanced by increasing the ratio of the size difference to amplicon length. In this study, we have developed two sets of highly polymorphic InDel markers for polyacrylamide gel electrophoresis and agarose gel electrophoresis so that researchers can determine which InDels may be useful, depending on their specific programs, by downloading our data without any limitations. In summary, the InDels with a high amount of polymorphisms and a large size difference will be useful as density markers of genome variation for marker-assisted mapping of important rice traits as well as for rice breeding, and the data generated in this study will be a valuable resource for rice improvement.

Moreover, the precise identification of InDels in sequence databases depends on the strategy and parameters used for data mining, thus these two points might be noteworthy. First, we adopted a computational strategy called e-PCR in this study; e-PCR can not only virtually simulate the PCR process but can also save time and reduce laboratory costs using *in silico* assays. Specifically, when developing new markers for mapping studies, e-PCR can be used to test potential primers in various ways before actually incurring the expense of oligonucleotide synthesis. Compared to variant identification software, such as samtools and GATK, which directly reads the map of the reference sequence, e-PCR utilizes hash-based string searching instead of whole sequence alignment. Second, the most important parameter for unique primer identification in e-PCR is fault tolerance. In this study, fault tolerance is set to three in e-PCR pipeline Step 2 to remove multiple loci as much as possible and increase the probability of the unique primers being aligned to reads from samples, while fault tolerance is set to one in e-PCR pipeline Step 3, given the tradeoff between InDel accuracy and SNP tolerance. Accordingly, the methods mentioned above greatly reduced the potential false positives in this article.

Finally, a much broader sample data set that was collected from publicly available genomic sequence information will also provide an opportunity to develop millions of InDels in representative wild and cultivated rice. The cultivated rice are broadly representative varieties with yield-related traits, quality-related traits and resistance-related traits, while the wild species of the genus Oryza contain a largely untapped reservoir of agronomically important genes for rice improvement. Thus, we developed genome-wide InDel polymorphism markers in both cultivated and wild rice that would greatly facilitate marker-assisted breeding and rice gene mapping.

## Conclusion

In this study, we developed a large amount of genome-wide and easily used InDel markers with a high amount of polymorphisms and density. The identification and validation of these InDel polymorphism markers among 1767 rice genomes will provide molecular markers for genetic study, such as gene cloning and association analysis, as well as marker-assisted selection in breeding.

## Methods

### Data Sources of Rice Genomic Sequences

We downloaded the genomic sequence data of two rice cultivars, Nipponbare (MSU version Release 7) and 9311, from the websites http://rice.plantbiology.msu.edu/index.shtml and http://rice.genomics.org.cn/rice/link/download.jsp. The 5′-UTR, CDS, 3′-UTR, exon, intron and intergenic sequences were provided by the annotation of the Nipponbare genome (http://rice.plantbiology.msu.edu/index.shtml). In addition, we also downloaded the re-sequencing data of 1765 rice varieties from the NCBI web site http://www.ncbi.nlm.nih.gov/sra?term=ERA000213, SRA023116, ERA081008, SRP026477, ERP000729, SRA051809, SRA036041, SRA036085, DRR000719, ERA009071, and SRA046411. All 1767 rice genotypes were composed of cultivated rice (*Oryza sativa* L.) and its wild relatives; thus, these inbred lines were selected to represent a diverse collection (Additional file 1: Table S1).

### Plant Materials and DNA Isolation

A panel of 20 rice cultivars, including 6 japonica-type rice cultivar (Nipponbare, yanjing2, yandao8, xudao3, huaidao13, wuyunjing3) and 14 indica-type rice cultivar (9311, zaomadao, SRSye, beixiang7, 60kang, 72gan, 955R, T116, sanpang76, R1318, nanhui511, guiyangai49, qingnongai, huangxinzhan), were used to validate InDel polymorphisms. Genomic DNA was isolated from 2-week-old seedlings using the modified CTAB (Cetyltrimethyl Ammonium Bromide) method (Murray and Thompson 1980).

### e-PCR Primer Design and Unique Primer Identification

**Fig. 5:**
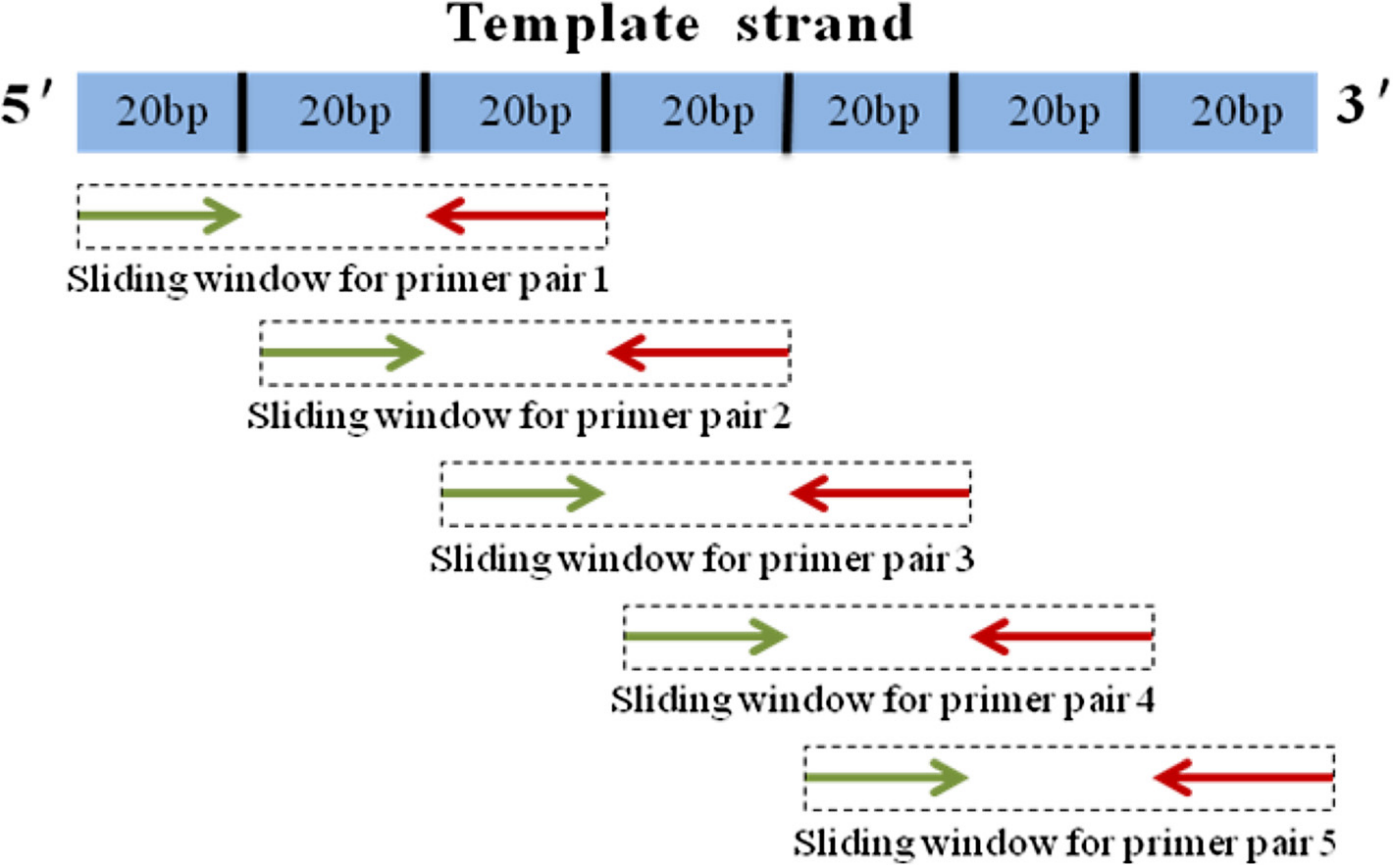
e-PCR primer design based on sliding window. The green arrow lines were the forward primers and the red arrow lines were the reverse primers

**Fig. 6:**
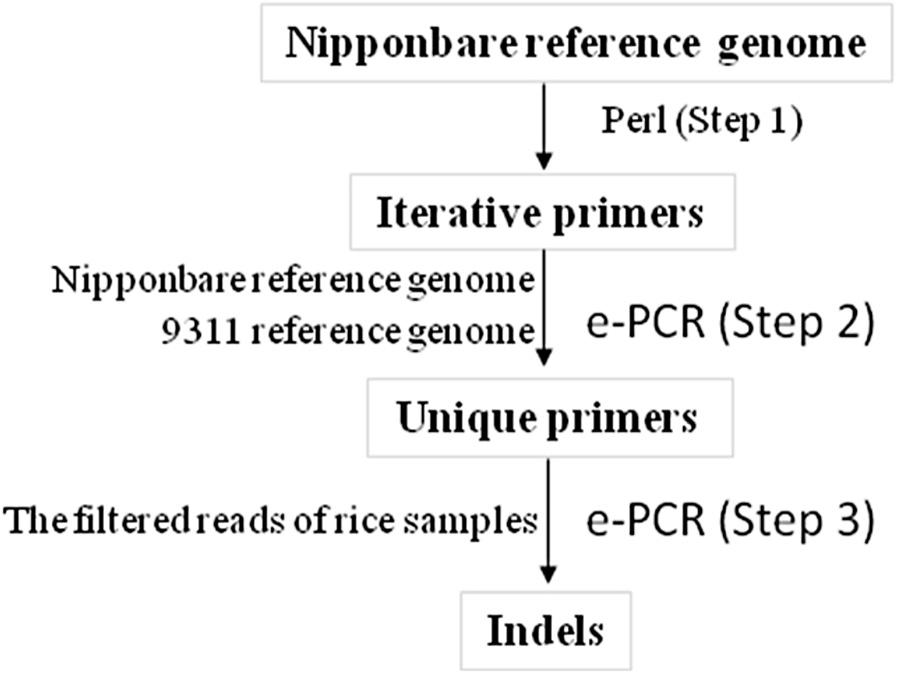
e-PCR Pipeline

Using the DNA sequences of the Nipponbare reference genome as the template, we designed e-PCR primers by a Perl script based on the sliding window technique. In the sliding window technique, a defined window is slid using each previous primer pair at a time along the template sequence, and then, a set of primer pairs are designed to cover the entire genome as much as possible (Yang et al. 2011). First, we extracted 20 bp from the first nucleotide of the template sequence as the forward primer, and after an interval of 20 bp, we extracted another 20 bp sequence as the reverse primer for the designed primer pair within the sliding window. Subsequently, the sliding window shifted based on the previous reverse primer, and the next forward primer was designed from the 20-bp upstream region of the previous reverse primer (Fig. 5). Thus, the remaining primers were designed in the same way, and this procedure continued until the entire template sequence was covered by all primer pair sets (Fig. 6 Step 1). After the sliding window process was performed, the sequences of primer pairs were aligned to the Nipponbare and 9311 reference genomes with Bowtie allowing up to three mismatches (Fig. 6 Step 2). Unique primers in both reference genomes simultaneously were also identified by a Perl script (Langmead et al. 2009).

### InDel Identification in Rice Varieties

We used NGSQC toolkit v2.3.3 to filter for high quality data (cutoff quality score of 20) (Patel and Jain 2012). Using the re-sequencing reads from 1765 rice varieties as the template, unique primer sequences were aligned with Bowtie, allowing up to one mismatch (Fig. 6 Step 3). If there were many e-PCR amplicon products for the same allelic locus, the most frequent one was selected by a Perl script, which was similar to the strategy for contig assembling in ABySS. The allelic diversity of each InDel with the e-PCR products in twenty or more than twenty rice genomes was assessed by PIC, which was defined as ${\mathrm{PIC}}_i=1-{\displaystyle {\sum}_{j=1}^n{\displaystyle {p}_{ij}^2}}$, where p_*ij*_ is the frequency of the *j*th pattern for the *i*th marker (Anderson et al. 1993).

### Analysis of Enriched Gene Ontologies

We used AgriGO (Du et al. 2010) at the default setting to examine the significant enrichment GO terms for each gene affected by InDels with PIC ≥ 0.5 and major allele differences ≥3 bp in the CDS region, and the gene annotation used in this study was obtained from MSU version Release 7.

### PCR Primer Design

To design primers for PCR validation, sequences with a total of 100 bp, including a 20-bp InDel region and flanking sequences of 40 bp for each side of the InDel region, were extracted. Primers were designed using Primer3 (Rozen and Skaletsky 2000), with a primer length of 20 nt to 28 nt and an optimal length of 23 nt, melting temperature (Tm) of 60 to 65 °C and an optimal temperature of 63 °C, PCR product length of 60 to 100 bp and ending with G- or C-rich at the 3′-end. Several primer pairs were designed for each InDel. The best primer pair was selected based on a similar Tm value with a GC content of 30-70 % and an optimal GC content of 50 %. All of the processes were carried out using custom Perl scripts.

### Experimental Validation of InDels

A set of 100 InDel markers (Additional file 6: Table S6) with PIC ≥ 0.5 and a 3–10 bp major allele difference in genomes of two inbred rice cultivars between Nipponbare and 9311 were randomly selected from the developed InDel markers for accuracy and polymorphism validation by the PCR technique. All 100 InDel markers were first used to amplify the genomic DNA of Nipponbare and 9311 for InDel accuracy validation. Then, we further detected their polymorphisms by PCR amplification of genomic DNA in a panel of 20 rice cultivars, including Nipponbare and 9311. For comparison purposes, 100 pairs of SSR primers were randomly selected (Additional file 7: Table S7), and the PIC value for each SSR marker was calculated using the formula described above.

PCR was performed in a 15-μL reaction volume containing 50 ng of template DNA, 1.5 μL of 10 × buffer (Mg^2+^), 2.0 μL of dNTP (2.5 mM), 100 nM of each SSR-primer, 2 U of Taq polymerase, and ddH_2_O. The DNA amplification protocol included an initial denaturation for 3 min at 95 °C, followed by 35 cycles of denaturation for 30 s at 95 °C, annealing for 90 s at 55 °C, and an extension for 90 s at 72 °C, with a final extension for 10 min at 72 °C. The reactions were performed in a C1000 thermal cycler (Bio-rad, Inc., Hercules, CA). The PCR products were subsequently separated in 6.0 % polyacrylamide gel and visualized using the silver-staining approach (Bassam et al. 1991).

## Additional files

Additional file 1: Table S1. Description of rice genotypes for e-PCR. (XLS 357 kb) Additional file 2: Table S2. e-PCR products of rice genotypes. (XLS 208 kb) Additional file 3: Table S3. InDels with major allele difference ≥3 bp. (XLS 19671 kb) Additional file 4: Table S4. InDels with major allele difference ≥8 bp. (XLS 10074 kb) Additional file 5: Table S5. Ontology analysis of genes affected by InDels with PIC ≥ 0.5 and major allele difference ≥3 bp in CDS region. (XLS 85 kb) Additional file 6: Table S6. Chromosomal position and primer sequence for 100 InDel markers. (XLS 37 kb) Additional file 7: Table S7. Chromosomal position and primer sequence for 100 SSRs. (XLS 33 kb) Additional file 8: Table S8. Allele number and polymorphic information content for InDel and SSR. (XLS 43 kb)

## Abbreviations

RFLP: Fragment length polymorphism
SSR: Simple sequence repeat
SNP: Single nucleotide polymorphism
InDel: Insertions-deletion
NGS: Next-generation sequencing
e-PCR: Electronic PCR
3′-UTR: 3′-untranslated region
5′-UTR: 5′-untranslated region
TSS_up_0.5 kb: 0.5 kb upstream of the transcription start site
TES_down_0.5 kb: 0.5 kb downstream of the transcription end site
CDS: Coding determining sequences
NCBI: National Center for Biotechnology Information
PIC: Polymorphism information content
Major allele difference: Size difference between the most and second most frequent alleles
InDel size difference: Size difference between the shortest and the longest InDel
Polymorphism rate: The proportion of InDels in the unique primer set
CTAB: Cetyltrimethyl Ammonium Bromide
